# Motor and Non-Motor Effects of Acute MPTP in Adult Zebrafish: Insights into Parkinson’s Disease

**DOI:** 10.3390/ijms26041674

**Published:** 2025-02-16

**Authors:** Niki Tagkalidou, Marija Stevanović, Irene Romero-Alfano, Gustavo Axel Elizalde-Velázquez, Selene Elizabeth Herrera-Vázquez, Eva Prats, Cristian Gómez-Canela, Leobardo Manuel Gómez-Oliván, Demetrio Raldúa

**Affiliations:** 1 Institute for Environmental Assessment and Water Research (IDAEA-CSIC), Jordi Girona, 18, 08034 Barcelona, Spain; niki.tagkalidou@idaea.csic.es; 2 Institute of Pesticides and Environmental Protection, Banatska 31b, 11080 Belgrade, Serbia; marija.stevanovic@pesting.org.rs; 3 Department of Analytical and Applied Chemistry, School of Engineering, Institut Químic de Sarrià-Universitat Ramon Llull, Via Augusta 390, 08017 Barcelona, Spain; ireneromeroa@iqs.url.edu (I.R.-A.); cristian.gomez@iqs.url.edu (C.G.-C.); 4 Laboratorio de Toxicología Ambiental, Facultad de Química, Universidad Autónoma Del Estado de México, Paseo Colón Intersección Paseo Tollocan, Colonia Residencial Colón, Toluca CP 50120, Estado de México, Mexico; gustavo.elizalde@icloud.com (G.A.E.-V.); herrera.v.selene@gmail.com (S.E.H.-V.); lgolivan74@gmail.com (L.M.G.-O.); 5 Research and Development Center (CID-CSIC), Jordi Girona, 18, 08034 Barcelona, Spain; eva.prats@cid.csic.es

**Keywords:** 1-methyl-4-phenyl-1,2,3,6-tetrahydropyridine, adult zebrafish, MPTP, Parkinson’s disease, prepulse inhibition, psychosis, turning difficulties, hypokinesia

## Abstract

Parkinson’s disease (PD), the second most common neurodegenerative disorder, is characterized by the progressive loss of dopaminergic neurons in the substantia nigra pars compacta, leading to motor and non-motor symptoms. The neurotoxin 1-methyl-4-phenyl-1,2,3,6-tetrahydropyridine (MPTP) has been extensively used in different animal species to develop chemical models of PD. This study aimed to evaluate the effects of acute exposure to MPTP (3 × 150 mg/kg, intraperitoneally) on adult zebrafish by assessing the neurochemical, transcriptional, and motor changes associated with PD pathogenesis. MPTP treatment resulted in a significant decrease in brain catecholamines, including dopamine, norepinephrine, and normetanephrine. Additionally, a trend towards decreased levels of dopamine precursors (tyrosine and L-DOPA) and degradation products (3-MT and DOPAC) was also observed, although these changes were not statistically significant. Gene expression analysis showed the downregulation of *dbh*, while the expression of other genes involved in catecholamine metabolism (*th1*, *th2*, *mao*, *comtb*) and transport (*slc6a3* and *slc18a2*) remained unaltered, suggesting a lack of dopaminergic neuron degeneration. Behavioral assessments revealed that MPTP-exposed zebrafish exhibited reduced motor activity, consistent with the observed decrease in dopamine levels. In contrast, the kinematic parameters of sharp turning were unaffected. A significant impairment in the sensorimotor gating of the ASR was detected in the MPTP-treated fish, consistent with psychosis. Despite dopamine depletion and behavioral impairments, the absence of neurodegeneration and some hallmark PD motor symptoms suggests limitations in the validity of this model for fully recapitulating PD pathology. Further studies are needed to refine the use of MPTP in zebrafish PD models.

## 1. Introduction

Parkinson’s disease (PD) is a progressive, neurodegenerative disease that affects 1.5% of the global population over the age of 65 years [1]. Despite years of focused research, current treatments are not fully effective and are generally associated with substantial side effects. Some of the motor disorders manifested in patients diagnosed with PD are bradykinesia, hypokinesia, impaired balance, rigidity, resting tremors, and smooth muscle spasms [2,3]. Axial and postural rigidity may contribute to turning difficulty, one motor hallmark symptom commonly found in PD patients [4]. All these symptoms are due to dopaminergic neuron loss in the substantia nigra pars compacta (SNpc), consequently leading to the disruption of dopamine input to the striatum, and finally motor impairment [5,6]. While the initial symptoms of PD are motor, the progression of the disease is frequently associated with psychiatric symptoms, with PD psychosis (PDP) occurring in 40–80% of patients [7]. The etiology of PD is complex and, along with genetic factors, various environmental contributors have been linked with an increased risk of disease manifestation. Extensive research over the years has revealed that several genes, like SNCA, PINK1, LRRK2, and PARK2, are involved in PD pathogenesis, with a number of other genes being under consideration and subject to further study [8,9,10]. Some of the non-genetic risk factors related to PD are exposure to pesticides and heavy metals, as well as to some naturally occurring substances (rotenone) or recreational drugs [2,8,9,10].

Animal models of PD are invaluable tools for understanding the mechanisms underlying the development of this pathology and for testing the chemical compounds either inducing or protecting against it. Zebrafish (*Danio rerio*) has emerged as a powerful model organism for studying PD due to its genetic tractability, high fecundity, and transparency during its early developmental stages. Moreover, zebrafish share a high degree of genetic and physiological homology with humans, particularly in the central nervous system. The dopaminergic system in zebrafish, including the enzymes involved in dopamine synthesis (e.g., tyrosine hydroxylase) and degradation (e.g., monoamine oxidase), as well as in the dopamine receptors, is highly conserved compared to humans [11,12,13].

Zebrafish models of PD can be broadly categorized into genetic and chemical models. Genetic models often involve the manipulation of PD-associated genes (e.g., PINK1, PARK2, or SNCA) using techniques such as CRISPR/Cas9 or morpholino knockdown. These models have provided valuable insights into the molecular mechanisms underlying PD, including mitochondrial dysfunction, oxidative stress, and impaired protein homeostasis [13,14,15]. On the other hand, chemical models of PD in zebrafish are typically induced by neurotoxins such as 6-hydroxydopamine (6-OHDA), rotenone, or MPTP, which selectively target dopaminergic neurons. These models are particularly useful for studying the behavioral and neurochemical aspects of PD, as well as for screening potential therapeutic compounds [16,17].

1-Methyl-4-phenyl-1,2,3,6-tetrahydropyridine (MPTP) is one of the most widely used neurotoxins for inducing PD-like symptoms in animal models. MPTP is a lipophilic compound that readily crosses the blood–brain barrier (BBB) [18] and is taken up by astrocytes, where it is metabolized by the enzyme monoamine oxidase B (MAO-B) into its active metabolite, 1-methyl-4-phenylpyridinium (MPP+) [19]. MPP+ is then released into the extracellular space and selectively taken up by dopaminergic neurons via the dopamine transporter (DAT) [20]. Inside dopaminergic neurons, MPP+ inhibits mitochondrial complex I of the electron transport chain, leading to a cascade of events, including ATP depletion, oxidative stress, and ultimately, dopaminergic neuron death in the substantia nigra pars compacta (SNpc) [5,13]. The resulting loss of dopamine input to the striatum manifests as motor dysfunction, mimicking the key features of PD.

While MPTP has been extensively used to model PD in rodents and non-human primates, its application in zebrafish is relatively recent and still evolving. Most studies in zebrafish have focused on the embryonic and larval stages, which are advantageous for high-throughput screening due to the small size and rapid development of the fish. However, adult zebrafish are increasingly being recognized as a valuable model for studying PD, particularly in the context of aging, which is a major risk factor for the disease. Adult zebrafish exhibit complex behaviors, such as shoaling, learning, and memory, which can be used to assess both motor and non-motor symptoms of PD [13,14].

Despite the growing interest in adult zebrafish models of PD, studies using MPTP in this context remain scarce and inconsistent in terms of methodologies. While most studies have reported significant hypokinesia in MPTP-treated zebrafish [3,11,12,21,22,23], other PD-like symptoms, such as turning disturbances, have not been thoroughly investigated. Turning difficulty is a relevant motor hallmark of PD that affects the kinematics of movement and is thought to result from axial rigidity and postural instability [4]. Additionally, it is still unclear whether acute MPTP exposure in adult zebrafish leads to the neurodegeneration of dopaminergic neurons or merely a transient decrease in dopamine levels [6]. Furthermore, no studies have yet explored the suitability of MPTP-based models of adult zebrafish for reproducing PD psychosis (PDP), a common non-motor symptom characterized by hallucinations and delusions [7].

In our study, we analyzed some components of the construction and face validity of an MPTP-based chemical model of PD developed in adult zebrafish. First, the effect of acute (3 days) exposure to this neurotoxic compound on the levels of dopamine and norepinephrine in the brain, as well as their main precursors and degradation products, was determined. Moreover, the expression level of the main genes involved in the synthesis (*th1*, *th2*, and *dbh*), metabolism *(mao* and *comtb)*, and transport (*slc6a3* and *slc18a2*) of catecholamines was also assessed to better understand whether the changes at the neurotransmitter level reflect regulation or neurodegeneration. Next, the potential hypokinesia of the exposed animals was evaluated by assessing basal locomotor activity in an open field test. For the analysis of turning difficulties, the kinematics of the acoustic startle response (ASR) were analyzed on the automated Zebra_K platform [24], since during ASR a sharp turn, known as a C-bend, is performed [24,25]. Finally, in order to assess the presence of PDP, the effect of MPTP exposure on prepulse inhibition (PPI) was determined as a measure of sensorimotor gating.

## 2. Results

### 2.1. Acute MPTP Exposure Reduces Brain Catecholamine Levels

**Figure 1:**
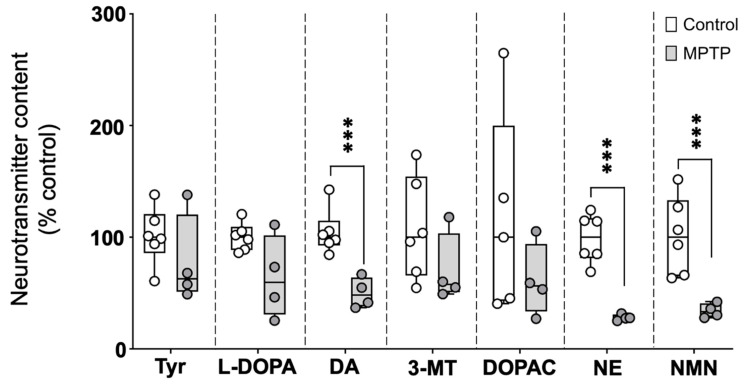
Profile of catecholaminergic neurotransmitters, precursors, and degradation products in the brains of control and MPTP-treated adult zebrafish. Results are presented as percentage of the control values. Boxplot representation of the content of each neurochemical expressed as percentage of the control, with the boxes indicating the 25th and 75th percentiles and the whiskers the maximum and minimum values, showing all data. *** *p* < 0.001. Tyr: tyrosine; L-DOPA: levodopa; DA: dopamine; 3-MT: 3-Methoxytyramine; DOPAC: 3,4-Dihydroxyphenylacetic acid; NE: norepinephrine; NMN: normetanephrine.

The catecholaminergic neurotransmitter profile was evaluated in brains of control and MPTP-treated fish, 24 h after the three injections. As shown in Figure 1 and Supplementary Table S2, the brain levels of the catecholamine neurotransmitters dopamine and norepinephrine (NE), as well as the NE metabolite normetanephrine, were significantly reduced in MPTP-treated fish. The observed reductions relative to the control values were 51.8% (IQR: 36.1–62.0%) for dopamine, 72.4% (IQR: 69.2–74.3%) for NE, and 66.9% (IQR: 59.4–71.6%) for normetanephrine [*U*(*N*_control_ = 6, *N*_MPTP_ = 4) = 0.0, *z* = −2.56, *p* = 0.009]. Although the levels of the dopamine precursors (tyrosine and L-DOPA) and metabolites (3-MT and DOPAC) were also lower in MPTP-treated animals compared to the controls, in this case, the differences were not statistically significant.

### 2.2. Acute MPTP Exposure Does Not Result in Dopaminergic Neuron Degeneration

In order to determine if the acute exposure to MPTP was producing degeneration in the catecholaminergic neurons, the expression of seven genes involved in the synthesis (*th1*, *th2*), degradation (*mao*, *comtb*, *dbh*), and transport (*slc6a3*, *slc18a2*) of dopamine was determined in the brains of the control and MPTP-treated fish. As shown in Supplementary Figure S1 and Supplementary Table S3, none of these genes showed significantly altered expression in the brain 24 h after the third MPTP injection.

### 2.3. Acute MPTP Exposure Induces Reversible Hypokinesia

**Figure 2:**
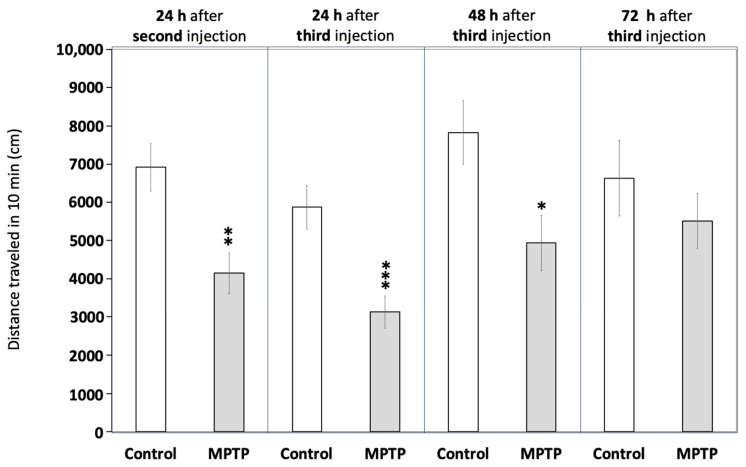
Acute exposure to MPTP leads to hypokinesia in adult zebrafish. Fish were videorecorded at different times for 10 min in the open field test paradigm and the total distance travelled was calculated. Data are reported as mean ± SEM (*n* = 23–24 for 24 h after the second and the third injections, *n* = 12 for 48 h after the third injection, and *n* = 8–12 for 72 h after the third injection). * *p* < 0.05, ** *p* < 0.01, *** *p* < 0.001; Student’s *t* test.

Figure 2 and Supplementary Table S4 demonstrate that MPTP exposure significantly reduced locomotor activity in the fish, an effect consistent with hypokinesia. Activity decreased to 57.9 ± 6.7% of the control values 24 h after the second injection (*t*(45) = 3.941, *p* = 0.0016) and further declined to 53.4 ± 7.2% after the third injection (*t*(44) = 3.808, *p* = 0.00043). To evaluate whether the observed hypokinesia was irreversible, as expected in PD, locomotor activity was also measured at 48 and 72 h after the final injection. Although still reduced compared to the control values, a mild recovery of locomotor activity was observed 48 h after the third injection (63.0 ± 9.2% of control values; *t*(22) = 2.615, *p* = 0.0158), with a more pronounced recovery evident at 72 h (83.1 ± 10.9% of control values; *t*(18) = 0.829, *p* = 0.418).

### 2.4. Kinematic Parameters During a Sharp Turn Remain Unaltered After MPTP Exposure

The acoustic startle response (ASR) in adult zebrafish is characterized by a ballistic, short-latency C-bend, providing a unique framework to evaluate whether MPTP exposure induces turning difficulties similar to those characterizing PD. As shown in Table 1 and Supplementary Table S5, no statistically significant differences were observed in any of the kinematic parameters between the control and MPTP-treated fish. However, the MPTP-treated group exhibited a non-significant trend towards a mild increase in the duration and magnitude of the C-bend. No evidence of loss balance was observed during the C-bend responses.

### 2.5. Acute MPTP Exposure Leads to Sensorimotor Gating Changes Consistent with Psychosis

**Figure 3:**
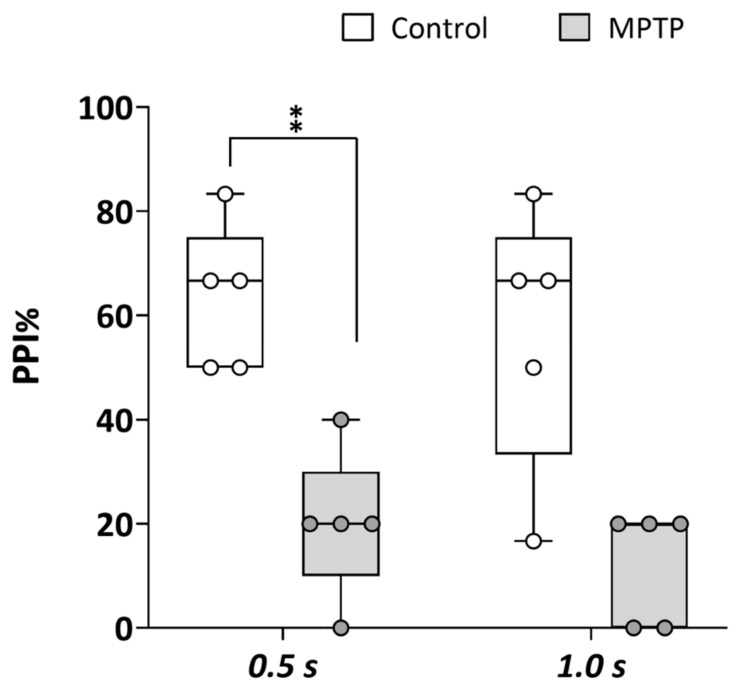
Acute exposure to MPTP leads to a strong decrease in prepulse inhibition (PPI) percentage. The assay was performed using the platform Zebra_K, with 2 different interstimulus intervals between the prepulse and the pulse: 0.5 and 1.0 s. Data are presented as boxplots, where the box indicates the 25th and 75th percentiles, the thin line within the box marks the median, and the whiskers represent the maximum and minimum values, showing all data. ** *p* < 0.01; Mann–Whitney U test. Data are from 2 independent experiments with 4–5 adult wild-type short-fin zebrafish in each experimental group in each experiment.

The prepulse inhibition (PPI) paradigm, a well-established measure of sensorimotor gating, is commonly used to assess disruptions in neural processes associated with psychosis. Therefore, to determine if the chemical model of PD developed in adult zebrafish by acute exposure to MPTP presented symptoms of PDP, PPI was analyzed in both the control and MPTP-exposed adult zebrafish using the automated platform Zebra_K. As shown in Figure 3, a significant decrease in PPI% was evident [*t*(8) = 3.776, *p* = 0.0054] in the MPTP-treated fish [20% (IQR: 10–30%)] with respect to the corresponding controls [67% (IQR: 42–75%)] when the interval between prepulse and pulse was 0.5 s. Although not statistically significant [*U*(*N_control_* = 5, *N_MPTP_* = 5) = 3.9, *z* = −2.015, *p* = 0.056], PPI% was also lower in the MPTP-treated fish [20% (IQR: 0–20%)] relative to the corresponding controls [50% (IQR: 25–75%)] when the interval between prepulse and pulse was 1 s.

## 3. Discussion

The current study demonstrates that acute MPTP exposure in adult zebrafish induces significant hypokinesia and decreases brain dopamine and norepinephrine levels. These findings are consistent with previous studies showing that zebrafish injected intraperitoneally with a single dose of MPTP exhibit reduced locomotor activity and swimming speed [3,11,22,26,27,28]. Studies that measured brain dopamine content in these models similarly reported significant neurotransmitter depletion [26,28]. Additionally, a reduction in mobility alongside decreased brain dopamine and norepinephrine levels has been observed in adult zebrafish 24 h after the intramuscular injection of MPTP [23].

Interestingly, in the study by Anichtchik et al., the authors found that despite the observed significant decrease in brain catecholamines 24 h after injection, no effects on catecholaminergic neurons were found using TH-immunohistochemistry, no changes in total TH protein were found by western blotting, and no signs of apoptosis or necrosis of the catecholaminergic neurons were evident using TUNEL staining and caspase 3 immunostaining [23]. These results are consistent with our finding that, despite the significant decrease in brain dopamine and norepinephrine observed 24 h after the last injection of MPTP, the expression of the genes involved in catecholamine metabolism (*th1*, *th2*, *mao*, *comtb*, and *dbh*) and transport (*slc6a3* and *slc18a2*) remained unaltered. These findings strongly suggest that the decrease in brain catecholamines found in adult zebrafish after acute MPTP exposure is not directly related to dopamine neuron degeneration. In fact, this study shows that the effect of MPTP on locomotion was fully recovered 72 h after injection, further supporting the absence of neurodegeneration in this model. However, it is important to consider that while whole-brain gene expression analysis provided a comprehensive overview of MPTP-induced effects, future studies incorporating region-specific morphological analyses and apoptotic markers could further clarify the extent of neuronal loss. Moreover, future studies should also include longer observation periods to provide additional insights into the temporal dynamics of MPTP-induced effects.

The transient nature of these effects may be attributed to the unique regenerative capabilities of the zebrafish brain. Zebrafish are increasingly used as translational neuro-regeneration models, as their stem cell activity is able to re-activate, even in adults, the molecular programs required for a neuronal regenerative response [29,30]. This remarkable neurogenic and repair capacity could partially counteract the damage caused by MPTP, allowing for the recovery of neurotransmitter levels and locomotor function.

While the main mechanism of action of MPTP primarily targets dopaminergic neurons [5,31], exposure to this chemical can also affect other neurotransmitter systems, including norepinephrine and serotonin, due to the shared reliance on mitochondrial function and the potential for cross-talk between neurotransmitter pathways [32,33]. The observed decrease in norepinephrine levels in our study may result from the shared vulnerability of noradrenergic neurons to mitochondrial dysfunction and oxidative stress, as these neurons also express the norepinephrine transporter (NET), which can uptake MPP+ [34]. Additionally, the interplay between dopamine and norepinephrine systems in regulating locomotor activity and stress responses may contribute to the hypokinesia observed in MPTP-treated zebrafish [5,6].

One important point to discuss about the observed hypokinesia in this model is the fact that either dopamine receptor antagonists or a decrease in dopamine levels decrease locomotor activity in mammals and fish [35]. Therefore, a phenotype consisting of reduced brain levels of dopamine with concomitant hypokinesia is not specific to PD, as it is a common finding in animals exposed to different neuroactive and neurotoxic compounds that decrease dopamine levels [36,37]. However, the transient nature of MPTP-induced hypokinesia in zebrafish, coupled with the absence of neurodegeneration, suggests that this model may be particularly useful for studying the early stages of dopamine depletion and the compensatory mechanisms that prevent neuronal loss.

There are aspects of human parkinsonism, such as bradykinesia and tremor at rest, occurring most prominently in appendicular muscle groups, that cannot be conveniently addressed in zebrafish [35]. However, the turning difficulty observed in PD patients is a symptom suitable to be determined in zebrafish, as it has been related to axial rigidity and postural instability. During turning, in contrast to the normal craniocaudal sequence observed in healthy adults, PD patients use an *en bloc* turning pattern characterized by the simultaneous onset of the axial segment rotation rather than the normal craniocaudal sequence [38]. To analyze the performance of a sharp turn in adult zebrafish after acute MPTP exposure, we determined, for the first time, the kinematic parameters during the C-bend of the acoustic startle response. Although no statistically significant differences were observed in any of the kinematic parameters, the MPTP-treated group exhibited a non-significant trend toward a mild increase in the duration and magnitude of the C-bend. Additional efforts should be made to assess this endpoint in genetic models of PD, as well as in chronic MPTP exposure paradigms, to better understand its relevance to PD-like symptoms.

Finally, in this study, MPTP-treated fish exhibited a significant decrease in PPI%, consistent with PDP in humans [7,39,40]. A similar reduction in PPI% has been reported in other animal models of PD. While this endpoint was not previously determined in MPTP-treated animals, a significant decrease in PPI% has been reported in 6-OHDA models of PD developed in rats [41,42,43,44] and mice [44]. Additionally, a genetic model of PD in mice with a reduced expression of Nurr1 exhibited a significant decrease in PPI% [45]. These findings suggest that MPTP-induced dopamine depletion may disrupt sensorimotor gating, a process that is also impaired in PD patients with psychosis. Further studies are needed to explore the mechanisms underlying this phenomenon and its potential as a biomarker for PDP in zebrafish models.

While our study provides valuable insights into the effects of acute MPTP exposure in zebrafish, several limitations should be acknowledged. First, the variability in MPTP exposure methods (e.g., intraperitoneal vs. intramuscular injection, dose, and duration) across studies may influence the observed outcomes, including the extent of hypokinesia and neurotransmitter depletion. Standardizing these parameters could enhance the reproducibility and comparability of results across different laboratories. Additionally, the transient nature of MPTP-induced effects in zebrafish, while useful for studying early compensatory mechanisms, may limit the model’s applicability to the chronic neurodegenerative processes seen in human PD. Future studies should explore chronic MPTP exposure paradigms or genetic models to better mimic the progressive nature of PD.

The findings from this study have important implications for understanding the early stages of dopamine depletion and the compensatory mechanisms that prevent neuronal loss. Zebrafish, with their remarkable regenerative capabilities, offer a unique platform to identify molecular and cellular targets that could be harnessed to promote neuroprotection or regeneration in human PD. Moreover, the observed decrease in PPI% in MPTP-treated zebrafish aligns with the sensorimotor gating deficits seen in PD patients with psychosis, suggesting that this model could be used to explore the underlying mechanisms of PD-related psychosis and to screen potential therapeutic interventions. Further research should focus on validating these findings in other animal models and exploring their relevance to human PD pathology and treatment strategies.

## 4. Material and Methods

### 4.1. Chemicals

1-Methyl-4-phenyl-1,2,3,6-tetrahydropyridine hydrochloride (MPTP; CAS 23007-85-4) was purchased from Sigma-Aldrich ((St. Louis, MO, USA; M0896, purity 100%) and dissolved in phosphate-buffered saline (PBS) at a concentration of 15 mg/mL before the start of the experiment.

### 4.2. Fish Husbandry

Adult zebrafish (standard length: 2.90 ± 0.01 cm) were obtained from Pisciber (Barcelona, Spain). Fish were maintained in a zebrafish recirculating system (Aquaneering Inc., San Diego, CA, USA) at the Research and Development Center (CID-CSIC) for two months prior to the experiments. Fish were reared in 2.8 L tanks with fish water (reverse-osmosis purified water enriched with 90 mg/L Instant Ocean^®^ [Aquarium Systems, Sarrebourg, France], 0.58 mM CaSO_4_ · 2H_2_O, and 0.59 mM NaHCO_3_) at 28 ± 1 °C under a 12/12 h (light/dark) photoperiod. They were fed twice per day with dried food (TetraMin, Tetra, Germany).

### 4.3. Experimental Design

All experimental procedures were approved by the Institutional Animal Care and Use Committees at the CID-CSIC (OH 1432/2023) and conducted in accordance with institutional guidelines under a license from the local government (agreement number 9820). The study adhered to the ARRIVE guidelines for reporting animal research [46].

#### 4.3.1. Animal Preparation and Anesthesia

Fish were randomly selected from breeding tanks (average weight: 0.4–0.45 g) and anesthetized using hypothermia to minimize stress during handling. This method is widely used in zebrafish studies due to its efficacy and minimal side effects [47]. After anesthesia, fish were positioned dorsoventrally for intraperitoneal (IP) injection.

#### 4.3.2. MPTP Administration

MPTP (1-methyl-4-phenyl-1,2,3,6-tetrahydropyridine) was dissolved in phosphate-buffered saline (PBS) at a concentration of 15 mg/mL. Based on preliminary dose–response experiments (doses: 80–200 mg/kg; injection frequency: 1–3–5 injections), the final experimental protocol was established. Fish received a total dose of 450 mg/kg body weight (bw), administered as three consecutive daily injections of 150 mg/kg bw. Control groups were injected with an equivalent volume of PBS. The IP injections were performed using a glass Hamilton syringe (10 µL) equipped with an ultrafine needle, delivering 10 µL of solution per gram of fish body weight.

#### 4.3.3. Housing and Environmental Conditions

Fish were housed in 2.8 L tanks maintained at 28 °C with a 12/12 h light/dark photoperiod, consistent with standard zebrafish housing conditions [48]. The tanks were cleaned daily, and fish were fed ad libitum to ensure optimal health throughout the experiment. Each experimental group was conducted in triplicate to ensure statistical robustness.

#### 4.3.4. Euthanasia and Tissue Collection

At the end of the experimental period, fish were euthanized via hypothermic shock in ice-cold water, a method approved for zebrafish euthanasia [49]. Brains were promptly dissected, flash-frozen in liquid nitrogen, and stored at −80 °C until further analysis.

### 4.4. Neurotransmitter Assessment

For the extraction of neurotransmitters, their precursors, and degradation products from adult zebrafish brain, the procedure described by Mayol-Cabré et al. [50] was followed. This procedure consisted of the homogenization of the samples using a TyssueLyser (Quiagen, Hilden, Germany) and subsequent centrifugation and filtration of the supernatant with a 0.22 μm Nylon filter directly into a chromatographic vial.

An internal standard mixture was added to all samples for the quantification of the internal standard calibration curve. Also, quality controls (QCs) were prepared by spiking samples with a native standard mixture at a concentration of 5 ppm of the compounds of interest. The QCs were used to evaluate the performed extraction procedure as well.

The solvents used for the extraction were acetonitrile (HPLC-MS grade), supplied by VWR chemicals (Leuven, Belgium); formic acid (LC-MS/MS grade) from Fisher Scientific (Loughborough, UK); and ammonium formate from Sigma-Aldrich (St. Louis, MO, USA). Ultra-pure water was obtained daily from the Millipore Milli-Q purification system (Millipore, Bedford, MA, USA).

Neurochemical analysis was performed by UHPLC-MS/MS. Separation and elution were achieved with the use of a BEH Amide column, and detection was performed in MRM mode with ESI+, guaranteeing specificity in detection and quantification.

To quantify the compounds of interest, a mixture of the following reference standards was used: tyrosine, L-DOPA, dopamine, 3-metoxytyramine (3-MT), 3,4-dihydroxyphenylacetic acid (DOPAC), norepinephrine (NE), and normetanephrine. These standards were purchased from Sigma-Aldrich (Steinheim, Germany). Moreover, an internal standard mixture containing DL-norepinephrine-d6 (NE-d6), 3-methoxytyramine-d4 hydrochloride (3-MT-d4), and dopamine-1,1,2,2-d4 hydrochloride (DA-d4) was also used in the quantification. These standards were purchased from Sigma-Aldrich and Toronto Research Chemicals (TRC, Toronto, ON, Canada).

### 4.5. Gene Expression Analysis

Total RNA was extracted from the whole brains of control and MPTP-exposed adult zebrafish (3 × 150 mg/kg fish bw, 24 h after the last injection) using Trizol Reagent (Invitrogen Life Technologies, Carlsbad, CA, USA), as described elsewhere [51]. RNA concentration was determined by spectrophotometric absorption in a NanoDrop™ ND-8000 spectrophotometer (Fisher Scientific). After DNase I treatment (Ambion, Austin, TX, USA), the first strand of cDNA was synthesized from 1 μg of total RNA using a First Strand cDNA synthesis Kit (Roche Diagnostics, Mannheim, Germany) and oligo(dT), according to the manufacturer’s instructions.

Changes in the expression of the target genes were confirmed by qRT-PCR, performed in a LightCycler^®^ 480 Real-Time PCR System with SYBR Green PCR Master Mix (Roche Diagnostics, Mannheim, Germany). Cycling parameters were as follows: 95 °C for 15 min followed by 45 cycles of 95 °C, 10 s, and 60 °C, 30 s.

Nine biological replicates were analyzed for each group, and three technical replicates were run in parallel for each individual sample.

The primer sequences of the seven selected genes—monoamine oxidase (*mao*), tyrosine hydroxylases (*th1* and *th2*), dopamine transporter (*slc6a3*), dopamine-β-hydroxilase (*dbh*), catechol-O-methyltransferase b (*comtb*), and vesicular monoamine transporter (*vmat2*), as well as the reference gene 2-peptidylprolyl isomerase A (*ppiaa*)—are listed in Supplementary Table S1. The efficiency and specificity of all primers were checked before the analyses.

The mRNA expression of each target gene was normalized to the housekeeping gene *ppiaa*. The relative abundance of mRNA was calculated following the ΔΔCt method [52], deriving fold-change ratios from these values.

### 4.6. Neurobehavioral Assessment

All behavioral experiments were conducted in a room with controlled environmental conditions (28 °C, darkness) in the timeframe of 10–17 h. All fish were acclimated for one hour in the behavioral room prior to testing.

#### 4.6.1. Locomotor Activity

To evaluate potential hypokinesia in the MPTP-treated fish, the basal locomotor activity was determined using the open field test, as described elsewhere [36,53]. Locomotor assessment was performed 24 h after the second injection (total dose: 300 mg/kg bw) and 24, 48, and 72 h after the third injection (450 mg/kg bw). Briefly, fish were placed in the center of a circular arena that was uniformly illuminated and recorded for 10 min. Total distance traveled was determined by video tracking analysis using EthoVisionXT 16 (Noldus, Wageningen, The Netherlands).

#### 4.6.2. Kinematic Analysis of the Acoustic Startle Response

Kinematic parameters of the ASR in adult zebrafish were determined using the Zebra_K platform, as recently described [24]. The effects of MPTP on sensorimotor gating were additionally addressed by assessing prepulse inhibition (PPI) on the same platform. Nine experimental arenas were recorded simultaneously (115 ms, 1000 fps) using a high-speed camera Photron Fastcam Mini UX100, (Photron Ltd., Tokyo, Japan). The analysis of the videos with the analysis software provided information on the main kinematic parameters of the initial C-bend: latency, duration (time between the latency and the moment of reaching the maximum bending), curvature (difference between the maximum body curvature during the C-bend and the initial curvature at the latency time), and maximal angular velocity. For PPI analysis, a low-intensity stimulus (1000 Hz, 10 μs, and 72.9 dB re 20 μPa), typically eliciting 0–10 % startle responses, was selected for the prepulse, and a startle-inducing stimulus (1000 Hz, 1 ms, 103.9 dB re 20 μPa) was selected for the pulse. A series of 5 prepulse stimuli (interstimulus interval (ISI): 120 s), then a series of pulse stimuli (ISI: 120 s), and finally, a series of 5 sequences of “prepulse + pulse” acoustic stimuli (ISI: 120 s) were delivered. For these sequences, the effect of different times between the prepulse and the pulse (0.5 and 1 s) was tested. The PPI percentage was calculated as described elsewhere [25].

### 4.7. Data Analysis

Statistical analysis was performed using IBM SPSS v29 (Statistical Package 2010, Chicago, IL, USA) and data were plotted with GraphPad Prism 9 for Windows (GraphPad software Inc., La Jolla, CA, USA). The normality of the data was assessed using the Shapiro–Wilk test. Descriptive statistics are presented as mean ± standard error (SEM) for parametric data, and as median and interquartile range (IQR) for non-parametric data. Data on locomotor activity (total distance travelled) and from the kinematic studies (latency, duration, curvature, and maximum angular velocity) were analyzed by unpaired t-test or the Mann–Whitney test with regard to the results of the normality distribution. Significance was set at *p* < 0.05.

## 5. Conclusions

In summary, our study demonstrates that acute MPTP exposure in adult zebrafish induces significant neurochemical and behavioral changes reminiscent of Parkinson’s disease (PD) pathology. Specifically, we observed a marked reduction in brain catecholamine levels, including dopamine, norepinephrine, and normetanephrine, alongside a trend toward decreased levels of dopamine precursors and metabolites. These neurochemical alterations were accompanied by reversible hypokinesia and a significant impairment in sensorimotor gating, as evidenced by the reduced prepulse inhibition (PPI) of the acoustic startle response (ASR), which parallels psychosis-like symptoms in humans. However, the absence of significant changes in the expression of genes involved in catecholamine metabolism and transport, as well as the lack of dopaminergic neuron degeneration, suggests that the acute MPTP model does not fully replicate the neurodegenerative aspects of PD.

While this model effectively captures some PD-like features, such as dopamine depletion and motor deficits, it falls short in reproducing hallmark motor symptoms like bradykinesia and tremor, which are specific to appendicular muscle groups in humans. Nevertheless, the acute MPTP-induced zebrafish model provides a valuable tool to study certain aspects of PD, particularly non-motor symptoms and early-stage neurochemical changes. Future studies should focus on refining this model, potentially through chronic MPTP exposure or combinatorial approaches, to better mimic progressive neurodegeneration and the broader spectrum of PD symptoms. Such efforts could enhance the utility of zebrafish as a model for PD research and drug discovery.

## Figures and Tables

**Table 1:** Kinematic parameters of the C-bend during the acoustic startle response in control and MPTP-treated adult zebrafish.

Parameter	Control (Median, IQR)	MPTP (Median, IQR)	*U* or *t	z	*p*
Latency (ms)	10 (10–11)	10 (10–11)	1232	1.486	0.137
Bending Duration (ms)	12 (9–14)	12 (11–15)	1270.5	1.707	0.088
* Curvature (°)	98.1 (81.3–109.9)	106.4 (94.5–120.0)	−1.929		0.057
Average Angular Velocity (°/ms)	8.3 (7.1–9.4)	8.4 (7.2–9.3)	1021.5	−0-250	0.802
Maximal Angular Velocity (°/ms)	18.0 (15.8–21.0)	17.5 (16.0–20.6)	1008	−0.356	0.722

* Normal distribution.

## Data Availability

The data supporting the findings of this study are available within the manuscript and its Supplementary Material file or will be made available from the corresponding author upon request.

## Supplementary Materials

The following supporting information can be downloaded at: https://www.mdpi.com/article/10.3390/ijms26041674/s1.
