# Supplementary material for: Motor and Non-Motor Effects of Acute MPTP in Adult Zebrafish: Insights into Parkinson’s Disease
Source: Int J Mol Sci. 2025 Feb 16;26(4):1674. doi: 10.3390/ijms26041674 (PMC11855887; doi:10.3390/ijms26041674)
Supplement: Supplementary file 1 [file ijms-26-01674-s001.zip › ijms-3457198-supplementary.pdf]

Supplementary Material

Motor and non-motor effects of acute MPTP in adult zebrafish: insights into Parkinson's disease

Niki Tagkalidou, Marija Stevanović, Irene Romero-Alfano, Gustavo Axel Elizalde-Velázquez, Selene Elizabeth Herrera-Vázquez, Eva Prats, Cristian Gómez-Canela, Leobardo Manuel Gómez-Oliván, Demetrio Raldúa

Supplementary Tables

Supplementary Table S1. List of primers used for qPCR

Gene	ZFIN Acc number	GenBank Acc number	Sequence		Amplicon length
comtb	ZDB-GENE-040724-164	NM 001083843	FW<br>RV	5'-ACTCGACCACAGCGTCTGCT<br>5'-AGCCCATTCGCGGTGTCTGC	108 bp
dbh	ZDB-GENE-990621-3	NM_001109694	FW<br>RV	5'-TGCAACCAGTCCACAGCGCA<br>5'-GCTGTCCGCTCGCACCTCTG	156 bp
mao	ZDB-GENE-040329-3	NM_212827.3	FW<br>RV	5'-GCAGTCAGAGCCCGAATC<br>5'-CACACCCATAAACTTGAGGAATC	106 bp
ppiaa	ZDB-GENE-030131-8556	NM_212758.1	FW<br>RV	5'-GGGTGGTAATGGAGCTGAGA<br>5'-AATGGACTTGCCACCAGTTC	179 bp
slc6a3	ZDB-GENE-010316-1	NM_131755	FW<br>RV	5'-AGACATCTGGGAAGGTGGTG<br>5'-ACCTGAGCATCATACAGGCG	151 bp
th1	ZDB-GENE-990621-5	NM_131149.1	FW<br>RV	5'-GACGGAAGATGATCGGAGACA<br>5'-CCGCCATGTTCCGATTTCT	95 bp
th2	ZDB-GENE-050201-1	NM_001001829.1	FW<br>RV	5'-CTCCAGAAGAGAATGCCACATG<br>5'-ACGTTCACTCTCCAGCTGAGTG	110 bp
vmat2	ZDB-GENE-080514-1	NM_001256225.2	FW<br>RV	5'-TGGAGCTCTGCAGCTTTTTGTGC<br>5'-AACGCCGGCTCCAGCATAGC	159bp

Supplementary Table S2. Profile of catecholaminergic neurochemicals in the brain of control and MPTP-treated (3 x 150 mg/kg bw. i.p.) adult zebrafish. Concentrations of each chemical are presented in pg/mg brain.

	Tyrosine	L-DOPA	Dopamine	3-MT	DOPAC	NE	Normetanephrine
**Control 1**	67022.9	63.9	197.2	1806.8	797.8	797.8	147.2
**Control 2**	54473.0	62.2	232.0	5377.9	737.0	737.0	123.2
**Control 3**	49449.5	55.2	128.3		443.5	443.5	64.2
**Control 4**	56601.2	93.3	92.3	3977.1	733.9	733.9	61.7
**Control 5**	58532.3	69.1	72.9	10537.1	549.5	549.5	90.4
**Control 6**	47763.3	66.9	138.5	1611.8	545.5	545.5	103.5
**MPTP 1**	40720.9	35.9	80.4	4191.4	203.9	203.9	40.9
**MPTP 2**	14080.8	43.8	157.5	2350.5	161.2	161.2	34.9
**MPTP 3**	25739.2	24.1	73.6	2125.0	176.2	176.2	27.0
**MPTP 4**	61788.2	27.2	65.5	1077.5	178.4	178.4	29.4

Supplementary Table S3. Expression profile of tyrosine hydroxilases (*th1* and *th2*), monoamine oxidase (*mao*). catechol-O-methyltransferase b (*comtb*), dopamine-β-hydroxilase (*dbh*), dopamine transporter (*slc6a3*), and vesicular monoamine transporter (*slc18a2*) in the brain of control (CN) and MPTP-treated (3 x 150 mg/kg bw. i.p) adult zebrafish. Housekeeping: *ppia*

	Cps								Log2 ΔΔCT values						
	ppia	***th1***	***th2***	***mao***	***comtb***	***dbh***	***slc6a3***	***slc18a2***	***th1***	***th2***	***mao***	***comtb***	***dbh***	***slc6a3***	***slc18a2***
**CN 1**	27.06	30.44	33.77	29.05	31.93	31.20	33.83	32.34	-0.2252	-0.0333	-0.8139	0.0269	0.8443	0.0848	0.0956
**CN 2**	23.31	27.62	29.78	24.90	28.90	29.28	30.14	29.53	-1.1619	0.2033	-0.4272	-0.6998	-0.9991	0.0181	-0.8511
**CN 3**	24.09	27.71	29.30	25.90	29.06	29.29	30.86	29.62	-0.4719	1.4633	-0.6439	-0.0698	-0.2191	0.0815	-0.1544
**CN 4**	24.77	28.71	31.41	26.04	29.96	30.17	32.24	30.64	-0.7885	0.0267	-0.1039	-0.2965	-0.4257	-0.6252	-0.5011
**CN 5**	26.03	28.50	33.68	27.31	31.53	30.99	32.67	31.55	0.6848	-0.9767	-0.1139	-0.5998	0.0209	0.2115	-0.1478
**CN 6**	26.91	29.86	34.57	28.01	31.40	31.88	35.30	32.82	0.1981	-0.9933	0.0694	0.4035	0.0009	-1.5452	-0.5378
**CN 7**	24.47	27.71	30.29	25.61	29.59	30.00	31.41	30.08	-0.0852	0.8617	0.0328	-0.2148	-0.5441	-0.0835	-0.2261
**CN 8**	27.06	29.58	36.07	27.05	31.47	30.96	32.31	30.96	0.7515	-2.2167	1.2994	0.6069	1.1976	1.7181	1.6006
**CN 9**	25.72	27.77	30.73	26.19	29.77	30.57	32.43	30.37	1.0981	1.6650	0.7011	0.8435	0.1243	0.1398	0.7222
**MPTP1**	24.55	28.17	33.64	26.87	30.16	30.52	33.43	31.83	-0.3469	-2.2917	-1.0222	-0.5881	-0.8674	-1.9102	-1.7761
**MPTP2**	26.93	30.87	34.11	27.99	31.34	32.58	34.78	33.30	-0.6435	-0.3583	0.2544	0.6352	-0.5307	-0.8535	-0.8494
**MPTP3**	27.30	30.72	32.48	29.07	31.72	33.00	38.58	31.93	-0.0885	1.6767	-0.4239	0.6569	-0.5457	-4.2485	0.9222
**MPTP4**	24.42	28.03	30.36	25.05	29.12	30.08	30.80	30.18	-0.3269	0.8650	0.6778	0.3319	-0.5474	0.6065	-0.2461
**MPTP5**	27.03	30.32	34.63	29.07	32.27	32.39	33.90	32.69	-0.1419	-0.9267	-0.8706	-0.3431	-0.3791	-0.0219	-0.2878
**MPTP6**	23.44	26.60	27.03	24.52	28.66	28.40	29.55	28.59	-0.0085	3.0767	0.0894	-0.3265	0.0176	0.7315	0.2256
**MPTP7**	24.73	27.61	30.05	25.31	29.13	30.20	32.11	29.88	0.2681	1.3550	0.5861	0.4935	-0.4957	-0.5302	0.2272
**MPTP8**	24.99	28.04	30.73	26.15	29.58	30.48	31.76	30.21	0.0981	0.9300	0.0111	0.3035	-0.5157	0.0748	0.1572
**MPTP9**	24.17	**27.93**	29.47	26.40	29.91	29.81	31.03	30.14	-0.6085	1.3733	-1.0556	-0.8381	-0.6574	-0.0119	-0.5894

Supplementary Table S4. Total distance (cm) traveled by control (CN) and MPTP-treated adult zebrafish during 10 min in the Open Field Test.

	24 h after 2 injections		24 h after 3 injections		48 after 3 injections		72h after 3 injections	
	**CN**	**MPTP**	**CN**	**MPTP**	**CN**	**MPTP**	**CN**	**MPTP**
**Experiment 1**	11457	5916.04	10272	2144.05	11644.4	7573.58	9577.08	7845.81
	11445.5	7622.22	7784.6	5471.1	9429.78	6326.11	1994.51	6202.84
	8449.84	5121.86	7205.8	4533.66	10007.6	5575.54	6929.35	1504.26
	4483.68	6858.85	5150.56	3496.95	5551.1	4142.13	11346.2	5347.85
	9173.25	206.765	3490.72	2083.46	4456.72	2083.46	11335.2	5690.13
	7416.4	6810.54	5062.15	1485.7	6651.91	2656.02	6789.87	6880.86
	9096.61	3820.29	779.785	1363.66	8859.41	9626.02	4602.62	6947.76
	6565.92	3400.06	6418.05	426.496	7803.55	6340.5	946.571	3654.49
	8238.54	9530.59	2272.33	3173.06	5121.75	3625.98	5244.75	
	13265.5	5358.7	10914.8	2664.76	12077.8	3041.11	9943.07	
	5146.65	6744.8	3194.63	281.349	2922.58	6827.76	6516.81	
	8951.4	3955.79	8569.94	1034.96	9382.08	1362.59	4364.12	
**Experiment 2**	5675.27	3722.17	2249.45	3997.08				
	7517.75	995.825	8627.51	462.057				
	2425.48	4094.66	8290.66	5290.09				
	709.945	2282.33	3000.9	7597.6				
	1425.58	7361.5	1697.26	4834.35				
	4700	939.423	8981.55	4361.31				
	7400.25	2524.18	7421.97	5466.52				
	8354.26	2645.85	5078.52	3909.52				
	5196.97	1821.76	6305.51	1584.34				
	7258.97	598.637	7193.4	3185.02				
	3950	2995.88	4171.94					
	7868.69		6832.07					

Supplementary Table S5. Kinematic parameters of the C-bend during the acoustic startle response in control and MPTP-treated adult zebrafish

Latency (ms)		Duration (ms)		Curvature (°)		Angular velocity (average, °/ms)		Angular velocity (maxima, °/ms)	
Control	MPTP	Control	MPTP	Control	MPTP	Control	MPTP	Control	MPTP
11	10	11	11	100.23	106.39	9.11	9.67	15.85	19.68
10	11	48	12	120.81	96.25	2.52	8.02	28.69	20.95
11	12	11	14	103.13	118.47	9.38	8.46	17.78	18.75
12	11	10	13	94.45	111.15	9.44	8.55	16.17	18.21
10	11	9	89	79.78	120.93	8.86	1.36	15.7	24.95
10	12	14	11	113.53	96.15	8.11	8.74	16.61	16.85
10	10	39	17	148.62	119.2	3.81	7.01	17.06	18.26
10	11	8	15	69.66	118.47	8.71	8.94	19.48	17.53
12	10	12	12	133.88	114.32	11.16	9.53	22.75	18.39
10	10	90	11	108.87	95.85	1.21	8.71	166.82	16.9
13	11	8	9	71.38	83.71	8.92	9.3	17.61	16.53
10	11	14	13	100.67	108.07	7.19	8.31	16.01	16.75
10	10	13	12	92.67	97.06	7.13	8.09	16.8	16.68
10	12	10	7	70.76	65.34	7.08	9.33	12.49	12.82
10	10	10	12	68.39	124.32	6.84	10.36	15.66	24.92
11	10	13	13	162.42	98.43	12.49	7.57	68.99	15.35
9	10	12	89	92.87	123.04	7.74	1.38	20.56	20.76
10	10	9	11	87.41	99.37	9.71	9.03	20.06	16.62
10	10	10	12	85.32	125.66	8.53	10.47	15.52	18.08
10	13	15	9	87.17	71.67	5.81	7.96	13.29	21.67
11	10	8	14	65.03	113.76	8.13	8.13	20.88	16.05
12	10	12	65	97.85	132.38	8.15	2.04	24.64	17.07
12	10	88	10	75.67	101.57	8.13	10.16	146.03	16.02
10	11	15	11	121.94	94.51	8.13	8.59	16.54	15.95
10	12	11	12	111.06	82.21	10.1	6.85	22.65	21.58
11	14	12	7	101.02	59.04	8.42	8.43	18.48	20.59
12	11	10	9	107.24	87.21	10.72	9.69	21.59	18.44
11	11	12	41	82.91	171.45	6.91	4.18	12.13	22.32
10	11	14	13	110.98	120.01	7.93	9.23	15.02	16.44
11	13	13	26	107.56	131.97	8.27	5.08	13.92	14.73
11	9	11	12	108.16	91.23	9.83	7.6	19.65	14.42
10	10	13	16	101.34	161.71	7.8	10.11	21.02	21.16
9	12	16	12	107.98	100.93	6.75	8.41	14.33	14.86
10	10	8	10	69.62	82.49	8.7	8.25	19.78	14.78
11	10	8	11	66.45	101.72	8.31	9.25	16.32	25.07
8	13	12	14	87.95	100.06	7.33	7.15	15.9	11.96
10	10	9	39	88.99	87.44	9.89	2.24	19.38	20.57
10	8	8	13	84	117.27	10.5	9.02	21.38	21.69
9	11	14	20	96.25	153.05	6.88	7.65	14.49	16.38
10	10	14	11	164.67	76.72	11.76	6.97	18.02	15.65
10	10	8	12	107.62	125.79	13.45	10.48	20.79	17.51
10	11	12	15	112.32	116.19	9.36	7.75	17.95	14.24
14	9	9	18	59.12	107.02	6.57	5.95	16.43	19.73
10		9		104.45		11.61		18.41	
9		60		111.55		1.86		33.25	
9		11		98.06		8.91		20.04	
9		9		78.66		8.74		15.29	
9		55		123.17		2.24		30.82	
15		4		29.99		7.5		12.74	

Supplementary Figures

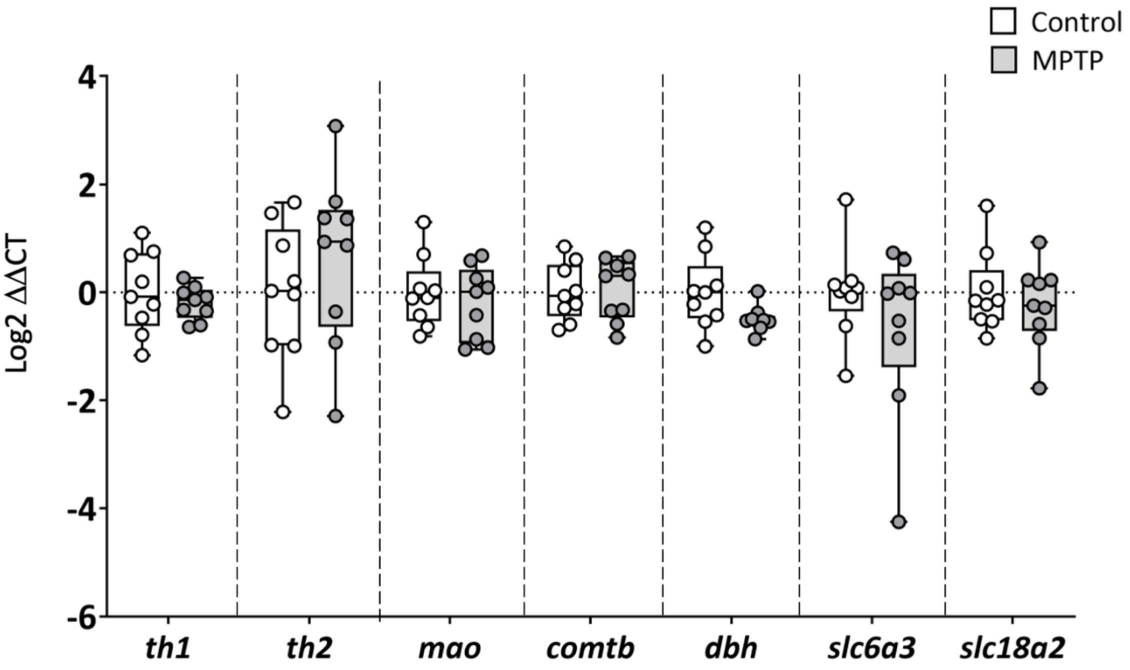

Supplementary Figure S1. No significant differences in the expression of catecholaminergic system-related genes 24 h after acute MPTP exposure (3 x 150 mg/kg bw, i.p.). Boxplot representation of ΔΔCt values, with the box indicating the 25th and 75th percentiles and the whiskers the maximum and minimum values and showing all data. The thin line within the box marks the mediane.
